# Elevations of α-fetoprotein in patients undergoing chemotherapy for pure testicular seminoma: a retrospective cohort study

**DOI:** 10.1186/s12885-025-13559-5

**Published:** 2025-02-11

**Authors:** Seán J. Costelloe, Jennifer D. Spencer, Kathryn Humphries, Daniel Stark, Elaine Dunwoodie

**Affiliations:** 1 Blood Sciences, Old Medical School, The Leeds Teaching Hospitals NHS Trust, Thoresby Place, Leeds, UK; 2 Department of Clinical Biochemistry, Cork University Hospital, Wilton, Co. Cork Republic of Ireland; 3 Department of Oncology, The Leeds Teaching Hospitals NHS Trust, Leeds, UK

**Keywords:** Α-fetoprotein, Outcome, Prognosis, Testicular seminoma

## Abstract

**Background:**

α-Fetoprotein (AFP) is conventionally absent in testicular classical seminoma (TCS). However, moderate AFP elevations can occur in TCS patients, as observed at this and other centres, which can be challenging to diagnostic and management practices.

**Methods:**

This retrospective cohort study considered AFP concentration in the context of germ-cell tumour diagnosis and characterisation at baseline (BL), disease status during chemotherapy, and long-term surveillance. The study considered patients with histologically diagnosed stage 1 TCS requiring chemotherapy over six years. For those with AFP above the reference interval at BL, histological imaging, case notes, and biochemical data were reviewed from BL to surveillance completion. Outcomes included AFP changes, diagnoses, therapy, disease progression, and death.

**Results:**

Of the 175 patients included, eight (4.6%) had elevated AFP at BL. Of these, two showed statistically but not clinically significant AFP changes during therapy, while six had moderate, stable AFP elevations with no changes in diagnosis during follow-up. During therapy, one patient developed metastases, and one died of causes likely unrelated to their TCS.

**Conclusions:**

Mild elevations of AFP in TCS may lead to diagnostic uncertainty or inappropriate management and investigation. However, AFP changes, alongside imaging, did not affect diagnosis, therapy, or follow-up at this centre for any of the patients examined. A subgroup of TCS patients has stable, moderate AFP elevations unrelated to tumour aetiology.

## Background

Testicular cancer (TC), most common in men aged 15–44 years (yr) old, accounts for approximately 1% of all male malignancies in the United Kingdom (UK) [1–7]. In the UK population, TCs are predominantly germ-cell tumours (GCTs) [4–7], and 45% of these, approximately 950 cases per year, are testicular classical seminomas (TCS) according to international classification systems [4, 6–9]. Tumour markers (TMs) aid the diagnosis, prognostication, monitoring of chemotherapy, and long-term surveillance of patients with GCTs [10], and elevations of α-fetoprotein (AFP) and human chorionic gonadotrophin (hCG) have good positive predictive value for GCT in the context of a testicular lump [11].

Since it is secreted by cells of yolk sac origin, conventional wisdom holds that TCS does not produce AFP at detectable concentrations [12]. However, moderately elevated AFP has previously been observed in patients with confirmed histological diagnoses of TCS at this centre. Indeed, AFP elevation in TCS is a more widely recognised phenomenon, with the potential for misdiagnosis and mismanagement of patients [13–16]. Moderate AFP elevations in TCS patients are proposed to result from a combination of non-tumoral sources [17, 18], undetected yolk sac elements [19, 20], chemotherapy effects [21], or analytical variability [22], underscoring the need for cautious interpretation and further research. Clarifying the clinical significance of moderate AFP elevations in TCS is challenging. There is no consensus definition for ‘moderate’ AFP elevation, and centres may use different analytical platforms. Without guidelines, clinical decision limits for AFP are often based on expert opinion. Since AFP elevations are unexpected in TCS, the significance of moderate AFP elevations remains unclear. More precise guidelines are needed to determine whether these elevations should impact diagnosis or treatment.

AFP is also commonly used in the surveillance of TC patients for recurrent disease following definitive chemotherapy and regular monitoring following treatment for advanced TCS, although evidence for the clinical benefit of this practice is currently lacking.

This retrospective cohort study considers AFP concentrations from baseline (BL) until the completion of planned surveillance in chemotherapy patients, where the histological diagnosis is TCS. This study examines AFP levels in TCS patients at baseline and during follow-up to assess their diagnostic value and clinical implications. This study aims to inform clinicians of the thresholds and trends in AFP concentration that warrant careful consideration in TC, where the histological diagnosis is TCS.

## Methods

### Study design

This retrospective cohort study was conducted at the Leeds Cancer Centre (LCC), a tertiary referral centre for TC. Patients diagnosed with stage 1 TCS between 1st January 2008 and 31st December 2013 were included in the analysis. Using unique patient identifiers, data for these men were collected retrospectively from electronic health records, laboratory information systems and the electronic patient record at LCC. The study aimed to evaluate AFP concentrations and outcomes during chemotherapy and subsequent follow-up.

### Patient population

Approximately 30 men per year requiring chemotherapy for stage 1 TCS are treated at LCC. TCS is defined as a seminoma confined to the testes, with no histological evidence of other forms of testicular cancer. The BL period was defined as the period from − 28 days up to and including the day of commencement of chemotherapy (Day 0). All eligible patients were retrospectively included in the study if they had confirmed stage 1 TCS either at BL or retrospectively; at least one AFP measurement at BL above the upper limit of the reference interval (ULRI); chemotherapy administered post-orchiectomy or for relapsed disease that was initially managed with surveillance.

Patients diagnosed with metastatic seminoma (stage IM or above), primary mediastinal, or retroperitoneal seminoma at baseline (by histology or imaging) were excluded from the study. These exclusions were implemented to maintain a focus on localised stage 1 testicular seminoma and ensure the integrity of AFP trend analyses. By excluding metastatic, mediastinal, and retroperitoneal seminomas, the study eliminates confounding factors related to advanced disease biology or non-gonadal origins, enabling a more accurate assessment of AFP concentration in the context of localised TCS. Patients are discussed using anonymised notations, such that “Patient X” is referred to as “P_X_” and so on.

### AFP measurement and statistical methods

AFP concentrations were measured using Siemens Centaur (pre-November 2018) and Atelica analysers at The Department of Blood Sciences of The Leeds Teaching Hospitals NHS Trust, and the agreement between the analysers is acceptable. The ULRI for AFP was 7 kIU/L, derived from a population of 780 individuals, encompassing 98.4% of the reference population (Siemens Healthcare). AFP concentrations between 8 and 14 kIU/L (1–2 * ULRI) were termed “moderate elevation” based on local expert consensus.

The reference change value (RCV) is a standard tool in laboratory medicine used to determine whether a change in a biomarker exceeds the combined effects of intraindividual biological variability (CV_i_) and assay imprecision (CV_a_). The RCV for AFP was calculated using the formula ($\:2.8*\:\sqrt{{CVi}^{2}*\:{CVa}^{2}}=2.8*\:\sqrt{{12.2}^{2}*\:{3.3}^{2}}=35.4\%$), where CV_i_ was 12.2% [23] and average CV_a_ was 3.3% for the AFP immunoassays. Changes exceeding the RCV are unlikely to be caused by variability or imprecision alone and may reflect a physiological or pathological process, such as a change in disease state and are termed “significant.”

The AFP assays performed satisfactorily in the UK National External Quality Assurance Scheme for the study period. AFP frequency and CV_i_ were compared between seminoma patients and reference populations [24]. Descriptive statistics were used to summarise data on patient demographics, AFP trends, and treatment outcomes. All statistical analyses were performed in Excel and Analyse-IT.

### Clinical assessments and follow-up

Each patient had AFP and human chorionic gonadotropin (hCG) levels measured at baseline, during chemotherapy, and periodically during post-treatment surveillance. Disease status was evaluated according to the Tumour-Node-Metastases (TNM) classification system for malignant tumours [25–27]. Patients entered a five-year surveillance program following the initiation of therapy, during which blood tests, chest X-rays, and computed tomography (CT) scans were conducted several times per year, as deemed clinically appropriate for each patient. Certain patients continued surveillance beyond five years upon the recommendation of the oncology team.

Patients were followed from the initiation of chemotherapy until June 2022. Data collected included patient age, chemotherapy regimens., histological and imaging findings at BL and during follow-up, and blood test results, including TMs. Outcomes considered were disease recurrence, all-cause mortality, and trends in AFP.

### Ethics statement

This study complied with UK data protection legislation, including the Data Protection Act 2018 [28], and the Caldicott principles [29]. All patient records and laboratory results were accessed solely by clinicians and scientists directly involved in patient care. Patients were treated according to standard care protocols, and no interventions or modifications to their care were made as part of this study. To ensure confidentiality, all data were anonymised before analysis by removing direct patient identifiers and assigning unique study identifiers.

According to the NHS Health Research Authority’s online decision-making tool for research ethics, a formal review by the NHS Research Ethics Committee (REC) was not required for this study, as it involved a retrospective review of fully anonymised patient data [30]. This determination aligns with UK guidelines for research ethics in studies involving anonymised health data.

To ensure compliance with UK data protection legislation and ethical standards, the authors consulted the Caldicott Guardian at Leeds Teaching Hospitals NHS Trust. The Caldicott Guardian confirmed that patient consent was not required as no additional data were collected and the study adhered to national legislation and guidelines. Furthermore, the Caldicott Guardian acknowledged the authors’ clear understanding of their responsibilities under the UK GDPR, the Data Protection Act 2018, and the Caldicott principles, affirming their compliance with these standards during the collection and processing of patient data.

## Results

### Patients included in the analysis

**Fig. 1:**
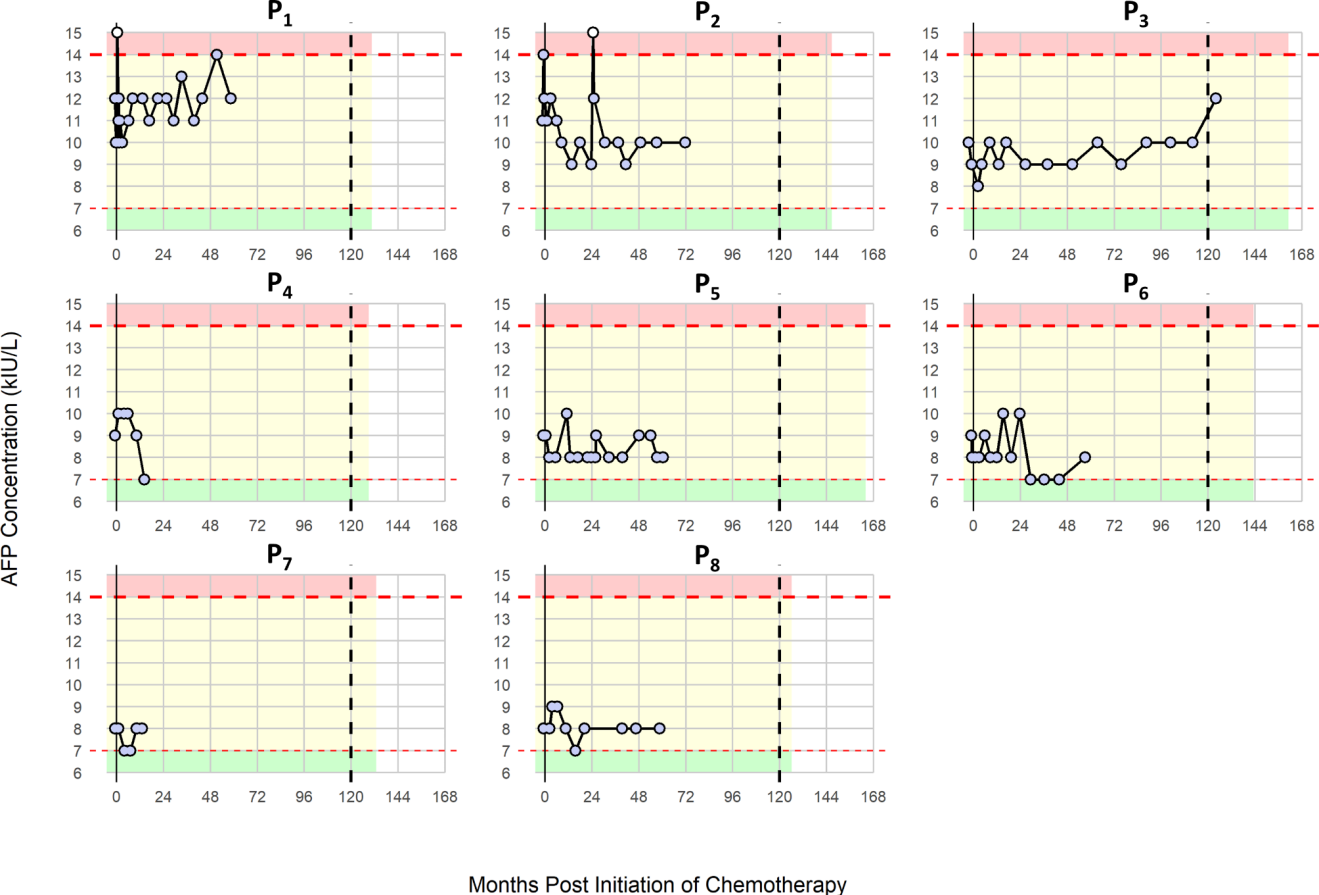
AFP concentrations over time for eight patients with elevated AFP levels at baseline. AFP timecourses for the eight individual patient cases during the baseline and surveillance periods reviewed at 10 years. The x-axis represents the months post-initiation of surveillance, while the y-axis indicates AFP concentration. AFP concentration ranges below the upper limit of the reference interval ((ULRI) < 7 kIU/L) are in green, the “moderate elevation” range (7–14 kIU/L) is in yellow, while the range twice the ULRI or higher (> 14 kIU/L) is in red. These shaded areas extend only as far as the individual patient follow-up, illustrating the varying surveillance durations for each patient. Horizontal dashed red lines indicate the upper limit of the reference interval (7 kIU/L) and twice the upper limit (14 kIU/L). Vertical solid black lines are drawn at 0 months, marking the start of surveillance, and dashed vertical black lines at 120 months, representing the 10-year follow-up point. Individual data points for AFP measurements are circles. Points filled in grey correspond to AFP concentrations within the green and yellow ranges (≤ 14 kIU/L), while white-filled points indicate AFP concentrations exceeding twice the upper limit of the reference interval (> 14 kIU/L). Each patient’s data is presented as a separate plot

Out of 175 men with an initial diagnosis of TCS and an AFP measurement at BL, 8 (4.6%) had confirmed stage 1 disease accompanied by moderately elevated AFP and were included as subjects in the analysis (Table 1). Subjects had a median age of 37 year at BL (interquartile range (IQR) = 12 year), and AFP was measured a median of 14 (IQR = 7.8) times for each patient in the BL and surveillance periods. Subjects had AFP measurements taken for a median period of 58.7 (IQR = 17.0) months (mo) following initiation of chemotherapy. Patient records were reviewed on 1st June 2022, allowing consideration of patient outcomes, and AFP concentrations (Fig. 1) over a median of 11.5 (IQR = 1.7) yr. The moderate elevations of AFP observed at BL did not affect their initial diagnosis or treatment.

### Chemotherapy regimens for subjects included in the study

In the subjects, chemotherapy regimens were as follows: carboplatin (AUC7) only in five patients; carboplatin (AUC7) with para-aortic radiotherapy in one patient; and bleomycin, etoposide and platinum (BEP) in one patient (Table 1).

**Table 1:** Details for testicular classical seminoma patients with AFP elevations at baseline in this study. The table summarises the characteristics of the eight men included in this study as summarised in the text. Abbreviations used are as follows: α-fetoprotein (AFP), Baseline (BL), Chemotherapy (CTX), Diagnosis (Dx), Follow-up (FU), Metastases (Mets), millimetre (mm), Past Medical History (Hx), Patient Identifier (PID), Tumour-Node-Metastases classification (TNM), years (yr)

PID	Age (yr)	Hx	Radiological Stage	Initial Dx	Final Dx	Mets during therapy	Size (mm)	Rete invasion	TNM stage	Necrosis	Days CTX to orchi	CTX regimen	CTX	BL AFP	Max AFP in FU	Min AFP in FU	Alive June 2022
**P**_**1**_	39	Seminoma contralateral testis 1995	IIIa	Seminoma	Seminoma	No	13	Yes	pT1	No	40	BEP 3 day	3-day BEP x 3	10	15	10	Yes
**P**_**2**_	43	COPD, gastric ulcer	I	Seminoma	Seminoma	No	25	Yes	pT1	No	42	CARBO (AUC) GCT	carbo (AUC 7) x 1	11	15	9	Yes
**P**_**3**_	45	Bilateral microlithiasis	I relapsed to IIa at 7 months	Seminoma	Seminoma	Yes	20	No	pT1	Yes (focal)	262	CARBO (AUC) GCT	carbo (AUC 7) x 1 + PA XRT	10	12	2	Yes
						(Stage 2 @ 6–7 months)											
**P**_**4**_	65	Undescended testes, Down’s Syndrome, chronic lung disease, left gynaecomastia, hypothyroidism, dementia	I	Seminoma	Seminoma	No	50	Yes	pT2	No	35	CARBO (AUC) GCT	carbo (AUC 7) x 1	9	10	7	No
**P**_**5**_	31	Testis surgery childhood, torsion and bilateral orchidopexy	I	Seminoma	Seminoma	No	30	No	pT1	No	43	CARBO (AUC) GCT	carbo (AUC 7) x 1	9	10	8	Yes
**P**_**6**_	29	No	I	Seminoma	Seminoma	No	24	Yes	pT2	No	50	CARBO (AUC) GCT	carbo (AUC 7) x 1	8	10	7	Yes
**P**_**7**_	35	Depression	I	Seminoma	Seminoma	No	40	Yes	pT1	No	35	CARBO (AUC) GCT	carbo (AUC 7) x 1	8	8	7	Yes
**P**_**8**_	32	No	I	Seminoma	Seminoma	No	60	Yes	pT1	Yes (extensive)	39	CARBO (AUC) GCT	carbo (AUC 7) x 1	8	9	7	Yes

### Follow-up of patients who had significant changes in AFP concentration during treatment

Two patients had significant changes in AFP concentration during treatment. Initially, P_1_ was treated as stage 3 disease due to a mediastinal mass on imaging. There was no retroperitoneal involvement, but the patients had a prior history of seminoma in the contralateral testis, for which para-aortic radiotherapy had been given. As a result, he was treated with three cycles of BEP chemotherapy. The mass persisted on completion of treatment, and when resected six months after his final dose of chemotherapy, histology was consistent with thymoma rather than metastatic TC. His disease was retrospectively recategorised as stage 1 disease, and he was included in our cohort. AFP increased significantly in P_1_ to a peak of 15 kIU/L on day 14, returning to 11 kIU/L on day 27 and remained moderately elevated until 58.7 mo. P_1_ tolerated chemotherapy well, and liver function tests were within normal limits during therapy.

P_2_ had a histological diagnosis of TCS with an AFP of 11 kIU/L pre-operatively. AFP increased to 14 kIU/L before the commencement of chemotherapy. At 24.7 mo, a single measurement of 15 kIU/L was taken. This reflected a significant change to his previous measurement. When repeated 14 d later, however, AFP had reduced to 12 kIU/L and remained between 9 kIU/L and 12 kIU/L until the end of the surveillance period at 72.1 mo. No additional investigations were required.

### Follow-up of patients with no significant changes in AFP concentration during treatment

In patients with moderate BL elevations of AFP, six (P_3-8_) remained moderately elevated, with no positive or negative changes in AFP concentration > RCV during follow-up. These patients were still considered to have TCS throughout the follow-up period.

Although P_3_ had moderately elevated AFP noted at BL, CT and histology confirmed classical TCS stage 1 without invasion and “no suggestion of non-semanomatous germ cell tumour (NSGCT)”. P_3_ was placed on intensive imaging and TM surveillance. A CT scan 6–7 mo later indicated stage 2 A metastatic disease, at which point P_3_ was commenced on carboplatin chemotherapy and sequential para-aortic radiotherapy. AFP remained moderately elevated at BL, and throughout treatment and follow-up, until the last measured AFP at 124.5 mo. In the remaining patients, no metastases were observed in these patients in the follow-up period, and one patient, P_4_, died. P_4_ had a history of undescended testes, Down’s Syndrome, chronic lung disease, hypothyroidism and dementia. The cause of death is not recorded on the electronic patient record at LCC but is not thought to be related to his TC.

### Biological variability for AFP and frequency of elevated AFP in Seminoma patients

For the seminoma patients included in this study, the CV_i_ for AFP was 10.3%, higher than the 4.5% quoted in reference databases [23]. The frequency of elevated AFP in the seminoma group was at least 4.1%, compared with 1.6% in the reference population. Thus, elevated AFP was at least 2.6 times more frequent amongst seminoma patients in this study than in the reference population.

## Discussion

### Patients with mild, stable elevations of AFP

In most subjects with moderate elevations of AFP at BL, AFP remained stable during follow-up, and none relapsed within the study period following treatment for seminoma. Recently, a study discussed “falsely elevated” AFP in patients with TC, who demonstrated moderate, stable elevations, not associated with disease or treatment, during prolonged follow-up [14]. The authors cautioned against interpreting moderate AFP elevations as evidence of embryonal carcinoma or yolk sac tumour and warned against inappropriate interventions. This study confirms, specifically for a TCS cohort, that some patients exhibit stable, moderate AFP elevations of unknown aetiology. However, these elevations were lower than those observed in the abovementioned study [14]. Consistent with previous assertions, this study reinforces that AFP elevations alone should not prompt alterations in diagnosis or chemotherapy but should be interpreted alongside other diagnostic measures.

### Patients with significant changes in AFP during chemotherapy in this study

Significant changes in AFP concentration were observed in just two patients during surveillance, although the maximum AFP observed for both men was 15 kIU/L. In neither case could this be correlated to disease status, therapeutic interventions, or any related comorbidity. However, one patient, initially treated for stage 3 disease, underwent a chemotherapy regimen identical to that used for NSGCT, in which AFP elevations are common. In this case, an AFP rise due to tumour lysis of a possible NSGCT component cannot be excluded.

### Strengths and deficiencies of this study

Strengths of this study include the fact that patient selection relied upon histological and staging information about patients at diagnosis, offering an unselected patient population.

Patient notes were retrospectively examined, and all study participants are now at least ten years from their initial diagnosis and chemotherapy. One patient died, and two were lost to follow-up, but the remainder completed the initial five years of formal surveillance activity. We, therefore, have robust data for this cohort about their long-term outcomes.

The authors identify the following deficiencies in this study: the study focuses on patients with moderate elevation of AFP at BL and does not consider patients with a change in AFP activity > RCV where the original BL measure was < ULRI. Further, since this is an observational study, there is a lack of uniformity in follow-up data, and the patient record could not be examined for an equal follow-up period in all patients.

### Defining “elevated AFP” in seminoma patients

Of importance is the definition of an abnormally elevated AFP. An assay-specific ULRI is used in this study, while other studies have defined higher cut-offs as clinically significant elevations of AFP based on local experience [13–16]. The authors suggest that clinical consideration should be given to all elevations of AFP > ULRI. Within our cohort, the highest level of AFP in patients not requiring a change in management was 15 kIU/L.

### Significance of AFP elevations in seminoma

Elevations of AFP in patients with histologically confirmed TCS may suggest several possibilities:

1. TC is classified incorrectly, and a non-seminoma element has been overlooked during the histological examination.
2. Comorbidities such as liver dysfunction, metastases, or tumour lysis syndrome.
3. An alternative AFP-producing tumour elsewhere.
4. Analytical factors, including assay reformulation or interference.
5. Moderate, stable AFP concentrations reflecting biological variability in the healthy population.

Understanding the aetiology of elevated AFP is vital since misinterpretation may lead to significant morbidity in these patients.

This study observed moderate elevations of AFP at BL and during surveillance in a small but significant proportion of men with new diagnoses of TCS. There was no apparent reason for raised AFP in these subjects, such as alcohol abuse, hepatitis, cirrhosis, biliary tract obstruction, and Fanconi anaemia, and there was no GCT relapse in these patients.

A key question is whether the patients with moderate elevations of AFP merely reflect biological variability in a healthy population. Although mild AFP elevations appear stable and independent of disease progress or chemotherapy, frequency in this group is more than three times that expected in the healthy population. Thus, they may not all represent physiological elevations. It is not immediately apparent if there is a mechanism for increased AFP associated with a seminomatous state, and the reasons for AFP elevation in this group remain obscure.

Interestingly, incipient yolk sac tumour (YST) micro-populations have been observed within seminomas. Forkhead box protein A2 (FOXA2) is considered a master regulator of YST formation, driving the reprogramming and differentiation of seminoma cells into YST-like cells through epigenetic mechanisms [31] and involving other transcription factors such as Sex-determining region Y-box 2 (SOX2) and SOX7, PReferentially expressed Antigen in Melanoma (PRAME), and Hepatocyte Nuclear Factor 1β (HNF1β) [32–36]. Although subpopulations of FOXA2-positive cells in pure seminomas are associated with increased tumour aggressiveness, potentially prompting therapeutic adjustment, they are not associated with altered AFP expression relative to seminomas lacking FOXA2 expression [37, 38]. Therefore, while it is striking that the prevalence of AFP elevations in the cohort described is similar to the ~ 5% prevalence of FOXA2-positive seminomas reported in prior studies [38], it is difficult to ascribe the patterns observed to this mechanism. However, the authors suggest that the role of transcription factors and epigenetics in reprogramming micro populations of AFP-producing seminomas warrants further investigation.

In this study, neither moderated elevations of AFP nor significant changes in AFP, albeit still within the moderately elevated range, led to any inappropriate patient management at this centre. Indeed, the AFP concentrations and changes did not impact the treatment or management of any of the subjects described. However, the risk remains, particularly at less specialist centres, that the AFP concentrations and changes observed in this cohort might confuse and delay appropriate diagnosis, treatments and surveillance of patients with TCS.

## Conclusions

In a large comprehensive retrospective study of clinical records in a regional cancer centre, the authors observe moderate and stable elevation of AFP in a significant number of new diagnoses of TCS. In this group, the biological variability appears higher, and elevated AFP is more frequent than in the normal population. The significance of stable AFP elevations remains unclear but does not relate to disease or therapy and did not alter patient management at any stage. However, centres should be vigilant to the phenomenon of AFP elevations in this patient group so as not to alter diagnoses, treatment or follow-up in a clinically inappropriate manner.

## Data Availability

The datasets used and analysed in the current study are available from the corresponding author upon reasonable request.

## Abbreviations

AFP: α-Fetoprotein
AUC: Area Under the Curve
BEP: Bleomycin, Etoposide, and Platinum
BL: Baseline
CT: Computed Tomography
CTX: Chemotherapy
CV: Coefficient of Variation
CV_a_: Analytical Coefficient of Variation
CV_i_: Intra-individual Biological Variability
D_x_: Diagnosis
FOXA2: Forkhead box protein A2
FU: Follow-up
GCT: Germ Cell Tumour
hCG: Human Chorionic Gonadotropin
HNF1β: Hepatocyte Nuclear Factor 1β
Hx: Past Medical History
IQR: Interquartile Range
LCC: Leeds Cancer Centre
Mets: Metastases
mm: Millimetre
mo: Months
NHS: National Health Service
NSGCT: Non-Seminomatous Germ Cell Tumour
PID: Patient Identifier
PRAME: PReferentially expressed Antigen in Melanoma
REC: Research Ethics Committee
RCV: Reference Change Value
SOX2: Sex-determining region Y-box 2
SOX7: Sex-determining region Y-box 7
TC: Testicular Cancer
TCS: Testicular Classical Seminoma
TM: Tumour Marker
TNM: Tumour-Node-Metastases classification
ULRI: Upper Limit of the Reference Interval
UK: United Kingdom
yr: Years
YST: Yolk sac Tumour

## References

[1] Cancer. registration statistics, first release, England, 2014. Office for National Statistics. 2014.

[2] Cooper A, Costelloe S. The clinical chemistry laboratory in the diagnosis and management of testicular cancer. Clin Lab Int. 2016 Apr-May;6–10.

[3] Hameed A, White B, Chinegwundoh F, Thwaini A, Pahuja A. A review in management of testicular cancer: single centre review. World J Oncol. 2011;2:94–101. 29147233 10.4021/wjon258w PMC5649662

[4] Bosl GJ, Motzer RJ. Testicular germ-cell cancer. N Engl J Med. 1997;337:242–54. 9227931 10.1056/NEJM199707243370406

[5] Hanna NH, Einhorn LH. Testicular cancer– discoveries and updates. N Engl J Med. 2014;371:2005–16. 25409373 10.1056/NEJMra1407550

[6] Horwich A, Nicol D, Huddart R. Testicular germ cell tumours. BMJ. 2013;347. 10.1136/bmj.f5526 24065428

[7] Barlow LJ, Badalato GM, McKiernan JM. Serum tumor markers in the evaluation of male germ cell tumours. Nat Rev Urol. 2010;7:610–7. 21068762 10.1038/nrurol.2010.166

[8] Eble JN, Sauter G, Epstein JI, Sesterhenn IA. Pathology and genetics of tumours of the urinary system and male genital organs. Lyon: IARC; 2004.

[9] Testicular cancer incidence statistics. Cancer Res UK. 2011.

[10] Metastatic malignant disease of unknown primary origin in. Adults: diagnosis and management. NICE guidelines [NG12]. London: National Institute for Health and Care Excellence; 2010. 39808017

[11] Bahrami A, Ro JY, Ayala AG. An overview of testicular germ cell tumors. Arch Pathol Lab Med. 2007;131:1267–80. 17683189 10.5858/2007-131-1267-AOOTGC

[12] Looijenga LH. Human testicular (non)seminomatous germ cell tumours: the clinical implications of recent pathobiological insights. J Pathol. 2009;218:146–62. 19253916 10.1002/path.2522

[13] Albany C, Einhorn L. Pitfalls in management of patients with germ cell tumors and slight elevation of serum α-fetoprotein. J Clin Oncol. 2014;32:2114–5. 24841979 10.1200/JCO.2014.56.0607

[14] Wymer KM, Daneshmand S, Pierorazio PM, Pearce SM, Harris KT, Eggener SE. Moderately elevated serum alpha-fetoprotein (AFP) among patients with testicular cancer may not be associated with residual cancer or need for treatment. Ann Oncol. 2017;28:899–902. 28137740 10.1093/annonc/mdx012

[15] Dieckmann KP, Anheuser P, Simonsen H, Höflmayer D. Pure testicular seminoma with non-pathologic elevation of alpha fetoprotein: a case series. Urol Int. 2019;99:353–7. 10.1159/000478706 28668957

[16] Nazer T, Ro JY, Amato RJ, Park YW, Ordonez NG, Ayala AG. Histologically pure seminoma with elevated alpha-fetoprotein: a clinicopathologic study of ten cases. Oncol Rep. 1998;5:1425–9. 9769381 10.3892/or.5.6.1425

[17] Schumacher S et al. Chemotherapy-induced hepatic dysfunction and its impact on tumor marker levels in testicular cancer patients. J Clin Oncol. 2015.

[18] Doehn C et al. Serum tumor markers in testicular cancer: clinical utility and pitfalls. Oncol Rep. 2006.

[19] Lutz A et al. Histological misclassification in seminoma with elevated alpha-fetoprotein: a need for careful re-evaluation. Eur Urol. 2018.

[20] Moch H, et al. WHO classification of Tumours of the urinary system and male genital organs. 4th ed. Lyon: IARC; 2016. 10.1016/j.eururo.2016.02.028 26996659

[21] Kawai K et al. Tumor lysis and transient alpha-fetoprotein elevation in seminoma patients undergoing chemotherapy. Jpn J Clin Oncol. 2014.

[22] Sturgeon C et al. National recommendations for tumor marker use in testicular cancer. Clin Chem. 2008.

[23] Aarsand AK, Fernandez-Calle P, Webster C, Coskun A, Gonzales-Lao E, Diaz-Garzon J et al. The EFLM Biological Variation Database.

[24] Braga F, Panteghini M. Generation of data on within-subject biological variation in laboratory medicine: an update. Crit Rev Clin Lab Sci. 2016;53:313–25. 26856991 10.3109/10408363.2016.1150252

[25] Brierley JE, Gospodarowicz MK, Wittekind C, editors. TNM classification of malignant tumours. 8th ed. Oxford: Wiley-Blackwell; 2016.

[26] Suspected cancer. Recognition and referral guidelines [NG12]. NICE. 2015.

[27] Albers P, Albrecht W, Algaba F, Boke-meyer C, Cohn-Cedermark G, Fizazi K, et al. Guidelines on testicular cancer. Eur Urol. 2015;68:1054–68. 26297604 10.1016/j.eururo.2015.07.044

[28] Data Protection Act. 2018, c. 12. 2018. https://www.legislation.gov.uk/ukpga/2018/12/contents/enacted

[29] National Data Guardian. The Caldicott Principles. 2020. https://www.gov.uk/government/publications/the-caldicott-principles

[30] Health Research Authority. Do I need NHS REC approval? https://www.hra.nhs.uk/approvals-amendments/what-approvals-do-i-need/

[31] Bremmer F, Hemmerlein B, Thelen P, et al. Reprogramming in germ cell tumours: FOXA2 as a master regulator of YST differentiation. J Pathol. 2023;259(2):123–35.

[32] Bremmer F, Thelen P, Hemmerlein B, et al. Micro-populations of incipient YST cells in seminomas: therapeutic implications. Histopathology. 2023;74(6):755–63.

[33] Bremmer F, Hemmerlein B, Thelen P, et al. FOXA2-positive subpopulations in seminomas. Clin Cancer Res. 2023;29(4):455–62.

[34] Gillis AJM, Stoop H, Hersmus R, et al. SOX2/SOX17 expression patterns in germ cell tumours. Mol Pathol. 2017;35(4):344–50.

[35] Looijenga LHJ, Gillis AJM, Stoop H, et al. PRAME and its role in germ cell tumour differentiation. Nat Rev Urol. 2022;19(8):473–88.

[36] Mostert MC, Stoop H, et al. HNF1β-mediated reprogramming in germ cell tumours. Oncogene. 2024;43(2):112–8.

[37] Gillis AJM, Hersmus R, Stoop H, et al. Epigenetic mechanisms in germ cell tumours: FOXA2 and HNF1β regulation. J Mol Med. 2024;35(5):453–62.

[38] Looijenga LHJ, Mostert MC, et al. Aggressive subtypes of germ cell tumours post-chemotherapy. Lancet Oncol. 2025;26(1):1–5.39756439

